# Minimally invasive facet restoration implant for chronic lumbar zygapophysial pain: 1-year outcomes

**DOI:** 10.1186/s13022-014-0007-5

**Published:** 2014-10-18

**Authors:** Hans-Jörg Meisel, Konrad Seller, Achim L?th, Karin B?ttner-Janz, Peter Stosberg, Alexander Moser, Larry E Miller, Jon E Block, Luiz Pimenta

**Affiliations:** 1 Center of Neurosciences, Department of Neurosurgery, BG-Clinic Bergmannstrost, Merseburger Strasse 165, Halle, Germany; 2 Spine Center Freiburg, Hornus Str. 18, Freiburg, D-79108, Germany; 3 Büttner-Janz Spinefoundation, Meinekestr. 6, Berlin, 10719, Germany; 4 Vivantes Klinikum, Landsberger Allee 49, Berlin, 10249, Germany; 5 Miller Scientific Consulting, Inc, 1854 Hendersonville Road, #231, Asheville 28803, NC, USA; 6 The Jon Block Group, 2210 Jackson Street, Suite 401, San Francisco 94115, CA, USA; 7 Instituto de Patologia da Caluna, Rua Vergueiro 1421-Sala 305, Sao Paulo, Brazil

**Keywords:** Facet, Glyder, Implant, Lumbar, Minimally invasive, Pain, Spine, Zygapophysial

## Abstract

**Background:**

The zygapophysial (facet) joint is the primary pain generator in one third of chronic low back pain cases. Current treatment options include temporarily palliative nonsurgical approaches, facet injections, radiofrequency denervation, and, rarely, lumbar arthrodesis. The purpose of this study was to assess the safety and effectiveness of a minimally invasive implant intended to restore facet joint function in patients with chronic lumbar facetogenic pain.

**Methods:**

This prospective, multi-center feasibility study enrolled patients with confirmed lumbar facetogenic joint pain at 1 or 2 levels who underwent at least 6 months of unsuccessful nonoperative care. Patients received a minimally invasive implant (Glyder® Facet Restoration Device, Zyga Technology, Inc., Minnetonka, MN) intended to restore facet joint function while preserving the native anatomy. Main outcomes included back pain severity using a visual analogue scale, back-specific disability using the Oswestry Disability Index (ODI), and adverse events adjudicated by an independent Clinical Events Committee.

**Results:**

Of 40 enrolled patients, 37 patients received the facet restoration implant and 34 patients had complete 1-year follow-up data available. Over the 1-year follow-up period, back pain severity decreased 41% and ODI decreased 34%, on average. Freedom from a device- or procedure-related serious adverse event through 1 year was 84%. Implant migration was observed in 3 patients and implant expulsion from the facet joint occurred in 3 patients. In total, 2 (5.4%) patients underwent implant removal through 1 year post-treatment.

**Conclusions:**

A minimally invasive facet restoration implant is a promising treatment option in select patients with chronic lumbar zygapophysial pain who have exhausted nonsurgical treatments, with therapeutic benefit persisting at 1 year follow-up.

## Background

The basic anatomic unit of the spine is a three-joint complex comprised of an intervertebral disc and paired zygapophysial (facet) joints. The main function of the facet joints is to limit movement in all planes of motion with a secondary role in weight bearing. Facet joints bear up to 25% of axial loads under normal conditions, and even greater loads in the presence of decreased disc height [1]. Results of animal [2],[3] and cadaveric [4]¿[6] studies support the hypothesis that repetitive strain accumulated over a lifetime increases the risk for facet arthropathy, similar to other synovial joints. In humans, degenerative changes in any component of the anatomic unit predictably lead to concomitant degenerative changes in the other components [7]¿[10]. Since the risk for disc height reduction increases with age, facet joint arthropathy is also relatively common in older adults. Although the facet joints are responsible for 1 in 3 cases of chronic low back pain, second in frequency only to degenerative disc disease [11], the role of the facet joint as a pain generator remains largely underappreciated due to difficulties in differential diagnosis and lack of consensus in optimal management of this condition.

Current treatment options include temporarily palliative approaches such as conservative care, medical management, corticosteroid injection, and radiofrequency denervation. Lumbar arthrodesis is typically reserved for patients with late-stage disease who have exhausted nonsurgical treatments although no convincing evidence exists to support this procedure for facetogenic pain [12]¿[14]. There is an obvious therapeutic gap for patients with chronic lumbar facetogenic joint pain who have unsuccessfully exhausted conservative treatments. The purpose of this feasibility study was to assess the safety and effectiveness of a minimally invasive implant intended to restore facet joint function in patients with chronic lumbar zygapophysial pain.

## Methods

### Ethics

All experimental procedures performed in this study were in strict accordance with a pre-defined protocol that was approved by all researchers and the local ethics committees (Santa Rita Independent Research Ethics Committee (Sao Paulo, Brazil) and Ethics Committee of the Medical Association of Saxony-Anhalt (Halle, Germany). Subjects provided informed consent before study participation.

### Study design

This prospective, multi-center, feasibility study was conducted at four investigative sites in Germany and Brazil under a common study protocol. The protocol specified enrollment of 40 patients with chronic facet joint pain at one or two levels. Patients were grouped based on surgical history. Group 1 had no previous surgery (other than discectomy) at the index level or adjacent level. Group 2 had previous surgery (other than discectomy) at the index level and/or adjacent level. Prior arthrodesis or facetectomy at the index level was contraindicated in both groups.

### Participants

Eligible patients underwent at least 6 months of nonoperative care and presented with radiographic (CT or MRI) evidence of facet disease at one or two levels from L1 to S1, verified by at least one positive (?20 mm reduction in back pain on a visual analogue scale, (VAS) intraarticular/periarticular facet injection in the last 6 months. Additional inclusion criteria included surgical candidates age ?18 years, pain severity ?60 mm (with predominantly back symptoms), and Oswestry Disability Index (ODI) ?40%. Main exclusion criteria were active infection, morbid obesity, pregnancy, grade 3 or 4 osteoarthritis [15], osteoporosis or severe osteopenia, tumor, cyst, fracture, spondylolysis, spondylolisthesis >3 mm, disc collapse ?50%, or neurological deficit at the index or adjacent level.

### Preoperative evaluation

Preoperative evaluation included demographics, medical history, back and leg pain severity, ODI, orthopedic examination, and imaging. The orthopedic examination included palpation of the index facet joint and loading of the joint in flexion/extension and lateral bending. Imaging evaluations included MRI or CT and 4-view x-rays within 6 months of study enrollment.

### Implant

The Glyder® Facet Restoration Device (Zyga Technology, Inc., Minnetonka, MN) is intended for treatment of patients with chronic facetogenic pain from L2 to sacrum, confirmed by diagnostic facet injection, due to degenerative facet joint disease or mechanical joint disease. The Glyder Device consists of a pair of PEEK-OPTIMA® wafers implanted in each facet joint. Each wafer has a smooth side to restore the sliding surface and a textured side that engages the articulating surface of the facet to prevent migration. Each implant also contains a platinum/iridium marker encapsulated within the body to enhance implant visualization under radiographic evaluation. The device is intended to relieve facet joint pain by restoring facet joint function, preserving the native anatomy without compromising future treatment options.

### Procedure

**Figure 1:**
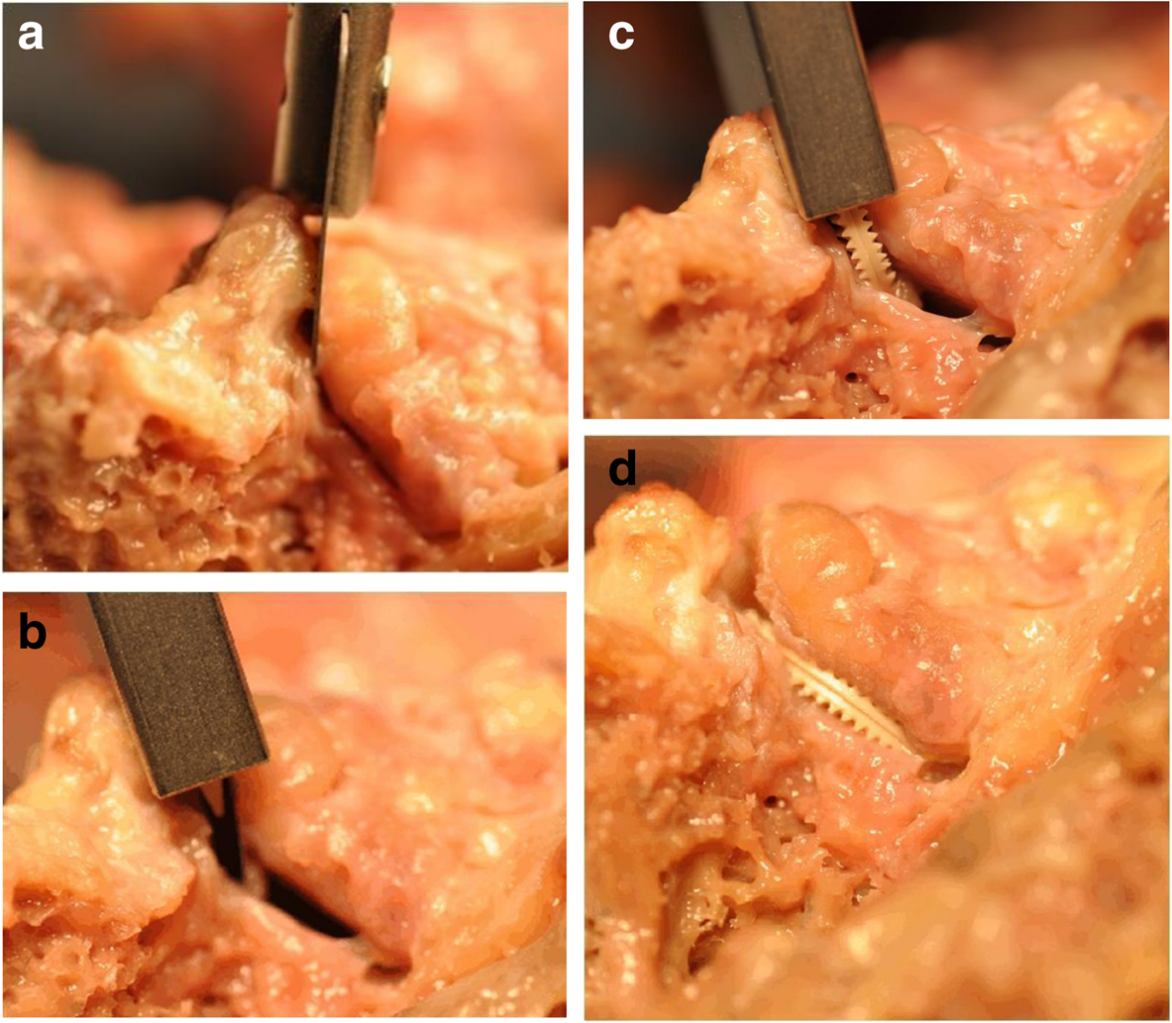
**Major facet restoration procedural steps including: a) joint access, b) distraction of the facet joint, c) implant insertion, and d) final implant position.** Facet capsule has been removed from cadaver for visibility, but is preserved during implantation.

Close inspection of preoperative imaging is mandatory to inform proper access trajectory and identify possible structures that may interfere with implantation such as large osteophytes, inadequate joint size such as <14 mm height and depth, or extreme posterolateral access angles that may result in occlusion by the iliac crest. The patient is positioned prone on a radiolucent spine table with lordosis maintained and the skin is prepped and draped. Under oblique fluoroscopic guidance, the correct level is identified and the posterolateral entry point is marked. A 2¿3 cm skin incision is made followed by blunt dissection to the facet joint capsule. The joint line is radiographically identified and a small (3¿5 mm) incision is made along the joint line on the posterior aspect of the facet capsule to open the facet joint (Figure 1a). The maximum amount of facet joint capsule should be preserved to maintain joint stability. Following slight distraction between both facet joint surfaces (Figure 1b), the Glyder implants are delivered with a dedicated tool through a cannula (Figure 1c). Following implant delivery (Figure 1d), direct and fluoroscopic visualization in two planes confirms correct positioning.

### Postoperative care

Patients were advised to avoid prolonged sitting, extreme bending or twisting, and lifting weights >5 kg during recovery. Typical recovery periods were 1 week to return to driving, 1¿3 weeks to return to a sedentary job, and 6 weeks for return to sport or physically demanding work. Patients were followed through hospital discharge and returned for follow-up visits at 6 weeks and 3, 6, and 12 months, which included a questionnaire, clinical examination and flexion/extension x-rays.

### Endpoints

The protocol defined effectiveness outcomes included back pain severity on a 0 to 100 mm VAS and back-specific disability using the ODI. Back pain clinical success was defined as a ?20 mm decrease in back pain severity compared to pre-treatment values [16],[17]. Back function clinical success was defined as an absolute decrease in ODI of ?15 percentage points compared to pre-treatment values [16]. Patient safety was assessed by recording all adverse events, regardless of severity or relationship to the device or procedure. An independent Clinical Events Committee (CEC) reviewed and adjudicated all events classified by the investigators as serious (SAE), device-related, and/or procedure-related.

### Statistical methods

All data were recorded on standardized case report forms and independently monitored for accuracy. Continuous variables were reported as mean?±?SD or median (min-max), depending on normality assumptions. Categorical variables were presented as n (%). Longitudinal outcomes were analyzed with repeated measures analysis of variance; values were reported as mean ±95% CI. Missing values accounted for <5% of VAS and ODI data and were conservatively imputed using the last observation carried forward (LOCF) technique [18]. Statistical analyses were performed using SPSS version 22 (IBM, Inc., Armonk, NY, USA).

## Results

A total of 40 patients (Group 1, n?=?24; Group 2, n?=?16) were enrolled at 4 investigative sites in Germany and Brazil between November 2009 and January 2013. One patient was discontinued from the study due to a negative diagnostic facet injection prior to the procedure. Two patients were discontinued when the procedure was aborted intraoperatively due to large osteophytes interfering with joint access. Ultimately, 37 patients were implanted with the facet restoration device. Two enrolled patients had grade 3 or 4 osteoarthritis at the index level (Table 1). Implants were routinely placed bilaterally at one (n?=?31) or two (n?=?4) levels. Two patients received a single implant at one level due to an inaccessible or non-implantable joint line at the contralateral facet. Both patients remained in the study and completed 1-year follow-up, one with no change in symptoms and the other with complete symptom relief. The minimally invasive procedure was associated with minimal blood loss and patients were typically discharged from the hospital in 2 days (Table 2). Complete 1-year follow-up data were available for 34 (92%) of 37 patients who received an implant. Reasons for missing 1-year follow-up visits included 2 patients who were lost to follow-up and one patient who withdrew from the study following device explant at 9 months.

**Table 1:** Baseline patient characteristics

**Variable**	**Value**
	***N?=?37***
**Male gender** ***, n (%)***	19 (51)
**Age** ***, yr***	52?±?13
**Body mass index** ***, kg/m***^*2*^	27?±?5
**Oswestry Disability Index***,%*	54?±?13
**Back pain severity** ***, 0¿100 scale***	77?±?13
**Leg pain severity** ***, 0¿100 scale***	45?±?38
**Facetogenic pain duration** ***, yr***	2 (0¿36)
**Facet joint osteoarthritis grade** ***, n (%)***	
???1	10 (27)
???2	25 (68)
???3	1 (3)
???4	1 (3)
**Previous nonsurgical treatments** ***, n (%)***	37 (100)
??-Injection*, n*	37
??-Medication*, n*	32
??-Physical therapy*, n*	28
??-Exercise*, n*	23
??-Chiropractic*, n*	9
**Previous back surgeries** ***, n (%)***	25 (68)
??-Fusion*, n*	9
??-Discectomy*, n*	7
??-Total disc replacement*, n*	7
??-Facet procedure*, n*	6
??-Disc nucleoplasty*, n*	2
??-Decompression*, n*	1
**Previous back surgery location** ***, n (%)***	
??-Any level (L1 to S1)	25 (68)
??-Index level	17 (46)
??-Adjacent level	18 (49)

Continuous data expressed as mean?±?sd or median (min-max).

**Table 2:** Procedure and hospitalization

**Variable**	**Value**
	***N?=?37***
**Index level** ***, n***	
??L2-L3	3
??L3-L4	9
??L4-L5	18
??L5-S1	11
**Levels with bilateral implants** ***, n (%)***	39 (95)
**Procedure duration** ***, min***	80 (30¿210)
**Procedural blood loss** ***, ml***	50 (0¿150)
**Hospital stay** ***, days***	2 (0¿6)

Continuous data expressed as median (min-max).

**Figure 2:**
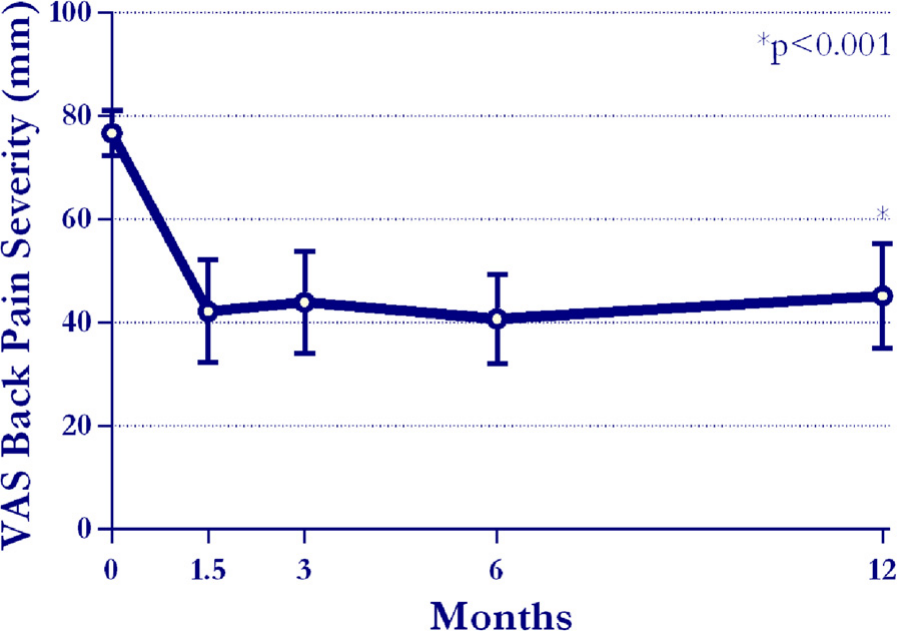
Change in visual analogue scale for back pain severity through 1 year.

**Figure 3:**
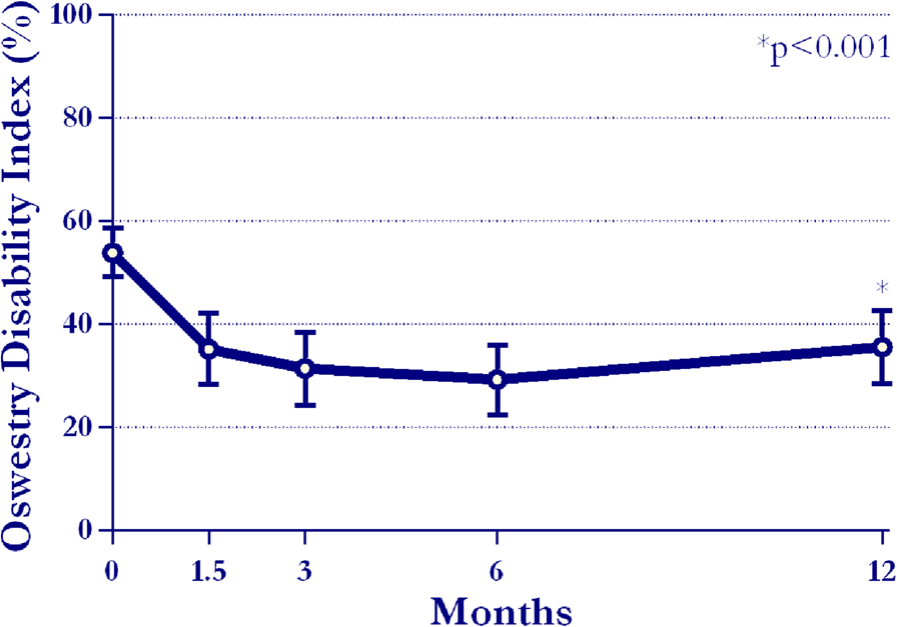
Change in Oswestry Disability Index through 1 year.

Back pain severity declined 35% on average through 6 week follow-up and remained stable through 1 year. Compared to pre-treatment values, back pain severity scores at 1 year were significantly lower (77?±?13 to 45?±?29, p?<?0.001) (Figure 2). At 1 year, 59% of patients reported at least a 20 mm reduction in back pain compared to pre-treatment. Back function improved by 34% on average during this period (54?±?13 to 36?±?21, p?<?0.001) (Figure 3). At 1 year, 50% of patients reported an absolute ODI decrease ?15 percentage points. No significant differences were noted between Groups 1 and 2 for any outcome.

**Figure 4:**
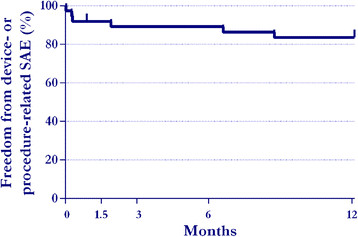
**Kaplan-Meier estimate of freedom from device- or procedure-related serious adverse event through 1 year.** SAE: serious adverse event.

Overall, 10 SAEs were reported in 8 (22%) patients. The CEC classified the events as related to the device (3), procedure (2), both (2), or neither (3) (Table 3). Freedom from a device- or procedure-related SAE through 1 year was 84% (Figure 4). Surgical wound infection occurred in two patients, one of which resulted in removal of the device in accordance with hospital policy. Implant migration defined as movement of the device in the joint was observed in three patients, two of which were defined as serious. One migrated device was removed due to the previously described wound infection. Implant expulsion demonstrated by evidence that the device exited the facet joint occurred in three patients, one of which required removal. In total, 2 (5.4%) patients underwent implant removal through 1 year post-treatment.

**Table 3:** Serious adverse events through 1 year

**Patient**	**SAE description**	**Days post**	**Procedure- or device-related**	**Reoperation**
a	Upper thoracic pain	136	None	No
b	Implant expulsion	201	Device	No
	Headache s/p myelography	288	None	
c	Surgical wound infection	58	Procedure	No
d	Implant expulsion	266	Device	Implant removal; pedicle screw fixation
e	Implant expulsion	9	Both	No
f	Lumbar spinal stenosis	321	None	No
g	Surgical wound infection	8	Procedure	Implant removal
	Implant migration	8	Both	
h	Implant migration	1	Device	No

## Discussion

The results of this prospective feasibility study suggest that facet restoration may be a viable treatment option in select patients with chronic lumbar zygapophysial pain, with therapeutic benefit persisting at 1 year follow-up. In comparison, the duration of therapeutic effect is typically 3 to 6 months with intraarticular corticosteroid injection [19]¿[21] and 3 to 12 months with radiofrequency denervation [22]¿[25]. Facet restoration implants may help to fill the therapeutic void in patients who have exhausted nonsurgical treatments.

This is only the second published study of minimally invasive lumbar facet restoration. A recent study by Van de Kelft [26] reported outcomes in 8 patients treated with the FENIX¿ facet resurfacing implant for lumbar zygapophysial pain. The results of this small trial were promising with a 72% reduction in back pain severity and 66% improvement in ODI at 1 year. One (12.5%) implant migration was observed at 2 years follow-up, which was treated with implant removal and posterior lumbar interbody fusion. Compared to the FENIX implant, a unique aspect of the Glyder Device is that no screws are required to maintain fixation. Instead, implant position is maintained by the textured surface gripping the facet articulating surface. This allows for a simple implantation procedure that preserves the native anatomy and, if needed, a straightforward explant. Additional studies are needed to clarify the comparative clinical utility of this implant design over the long term.

Five patients reported a clicking sensation immediately following implantation. In all cases, there were no adverse outcomes and the sensation eventually resolved without intervention. Presence of large osteophytes should be an absolute contraindication, as evidenced by two cases of unsuccessful implantation attempt in such patients. Although avoidance of implant migration is an intuitive concept, the clinical significance of this event is yet to be determined. Two patients with implant migration/expulsion underwent implant removal though it is unlikely that implant migration/expulsion in and of itself would elicit symptoms. Posterior implant expulsion should be benign since the device will simply imbed in the spinal musculature. Anterior migration is unlikely due to presence of the ligamentum flavum on the anterior portion of the facet joint. Nonetheless, implant migration is an untoward risk that can be mitigated with proper patient selection (e.g. adequate joint height and depth, proper facet osteoarthritis grade) and meticulous attention to device placement.

Limitations of this study included lack of a control group and a somewhat heterogeneous patient population. Arguably, use of ?20 mm improvement in VAS back pain severity as the criterion for a positive diagnostic injection in this study may be viewed as conservative with potential for false positives. Lessons learned from this feasibility study have led to modifications in implant design, implantation procedure, and patient selection.

## Conclusions

A minimally invasive facet restoration implant is a promising treatment option in select patients with chronic lumbar zygapophysial pain who have exhausted nonsurgical treatments, with therapeutic benefit persisting at 1 year follow-up.

## Competing interests

KB, LM and JB are consultants to Zyga Technology, Inc.

## Authors¿ contributions

HM, KS, AL, KB, PS, and LP participated in the design of the study in patient enrollment and treatment. LM performed the statistical analysis. LM and JB drafted the manuscript. All authors provided critical review of the manuscript draft and have given final approval of the version to be published.
